# A national-scale dataset for neighbourhood-level urban building energy modelling for England and Wales

**DOI:** 10.1038/s41597-026-07621-5

**Published:** 2026-06-26

**Authors:** Grace Colverd, Ronita Bardhan, Jonathan Cullen

**Affiliations:** 1 Department of Engineering, University of Cambridge, Cambridge, United Kingdom; 2 Sustainable Design Group, Department of Architecture, University of Cambridge, Cambridge, United Kingdom

**Keywords:** Civil engineering, Energy modelling

## Abstract

Buildings are significant contributors to global greenhouse gas emissions, accounting for 26% of global energy sector emissions in 2022. Meeting net-zero goals requires a rapid reduction in building emissions, both directly from the buildings and indirectly from the production of electricity and heat used in buildings. National energy planning for net zero demands both detailed and comprehensive building energy consumption data. However, geo-located building-level energy data is rarely available in Europe, with analysis typically relying on anonymised, simulated or low-resolution data. To address this problem, we introduce a dataset of **N**eighbourhood **E**nergy, **B**uildings, and **U**rban **La**ndscapes (NEBULA) for modelling domestic energy consumption for small neighbourhoods (5–150 households). NEBULA integrates data on building characteristics, climate, postcode area, housing density, environment, and socio-demographics, comprising 613,290 samples across England and Wales. It is designed to support evidence-based policymaking across housing, environment, and energy justice domains through integrated spatial-demographic analysis.

## Background & Summary

Urban building energy modelling (UBEM) is an important tool for understanding building stocks and their associated energy use. Modern statistical UBEM methods rely on large samples of building-level energy data and associated explanatory building and occupant factors (Ali *et al*.^1^). The energy data is typically collected from utility companies, or published by local governments. Privacy concerns surrounding building-level energy data and varying global regulations have led to a lack of open-access building-level energy datasets, as noted by Jin *et al*.^2^. The majority of open energy datasets are located in cities in the US, 22 out of a surveyed 33^2^. This concentration of data raises concerns about the potential overfitting of forecasting models to these open datasets (Kazmi *et al*.^3^), and limits the national planning of decarbonising building stocks. Of the open datasets reviewed, two datasets were from the UK. The first is the National Energy Efficiency Data Framework (NEED), an anonymised dataset released with representative data on buildings within the UK. The full NEED dataset comprises 4 million samples, including building and occupant characteristics, as well as energy consumption^4^. Anonymised building datasets like NEED do not enable fine-grained spatial analysis of energy consumption. Buildings within NEED are tagged with their region, but localised analysis is not possible. The second is the Energy Performance of Buildings Data, which provides access to the Energy Performance Certificates (EPCs) of buildings in England and Wales. EPCs are a standardised method of assessing the energy efficiency and environmental performance of a building. These certificates, mandated by the European Union in 2003, provide quantifiable data regarding a dwelling’s energy consumption and emissions^5^. The European Parliament mandate on EPCs allowed them to be calculated either through direct measurement of energy use or via methodological calculation^6^. EPCs have been mandatory for any house sold or rented in the UK since 2008 (DESNZ^7^). EPCs suffer from a noted performance gap where actual energy consumption differs from predictions (De Wilde^8^). Few *et al*.^9^ found that EPCS tend to over-predict primary energy intensity, with the over-prediction worsening for lower EPC bands (8% for band C vs. 48% for bands F and G), persisting even when matching assumptions around occupancy and heating styles. Furthermore, Jenkins *et al*.^10^ found that EPC ratings for the same building could vary by up to two bands across different assessments.

Closed-source building-level energy datasets also exist in the literature. In the UK, the 3DStock model exemplifies a closed-source approach. It links building-level energy data with Energy Performance Certificates (EPCs) along with other data sources to develop 3D building stock models for various areas, including London (Evans *et al*.^11^, Godoy-Shimizu *et al*.^12^). The potential privacy concerns of the building energy data mean that access to this model is tightly controlled.

The NEBULA (**N**eighbourhood **E**nergy, **B**uildings, and **U**rban **L**andscapes) dataset presented in this paper offers an alternative: a novel, privacy-preserving, geo-located, open source dataset for energy modelling in the UK at the neighbourhood level (5–150 households). We include domestic energy data for 2022, avoiding the challenges of simulated data or EPC unreliability. Working at the postcode level avoids privacy concerns and enables open-source publication. Our methodology is a true bottom-up methodology, deriving postcode-level attributes relating to the building stock, climate, housing density and socio-demographics. This dataset is targeted towards policymakers, researchers, and practitioners working in energy efficiency, net-zero, urban planning, energy justice and climate policy. The data has already been used to provide energy benchmarks for cities in the UK (Colverd *et al*.^13^) and for predictive modelling for retrofit assessment at the postcode level (Colverd *et al*.^14^).

## Methods

The following section describes the methodology for the dataset generation and the processes taken to derive the final dataset.

### Dataset Generation

The NEBULA dataset currently includes seven themes of variables: building stock, building typology, building age, region, housing density, climate and socio-demographics. These variables are derived from six datasets, open-source or accessible under academic licenses. Table 3 lists the input data sources and their licences. In total, NEBULA contains 232 variables (125 of which relate to socio-demographics, 94 to buildings, energy and environment and 13 to regional variables) and is in tabular format indexed by Postcode. The full list of variables and metadata (Column Name, Data Type, Category, Direct/Derived, Source, Description and Usage Notes) is provided alongside the dataset. A selection of the variables across the themes is given in Tables 1 and 2. The lookup tables used to link between data sources are given in Table 4. The following sections describe the variable creation process for each theme using a single postcode *PC* as an example.

**Table 1:** Selection of NEBULA Variable List in plain text, Part 1.

Building Stock	Typology
Count of All Buildings	Pct 2 Storeys Terraces w/ Rear Extension
Total Residential Building Footprint Area	Pct 3-4 Storey and Smaller Flats
Total Residential Building GEA	Pct Domestic Outbuilding
Total Residential Building GIA	Pct Large Detached
Count of Buildings with basements	Pct Large Semi-Detached
Total Basement GEA	Pct Linked and Step Linked Premises
Count UPRNS	Pct Planned Balanced Mixed Estates
Count Outbuildings	Pct Semi Type House in Multiples
Total Outbuildings GEA	Pct Small Low Terraces
Total Outbuildings Footprint area	Pct Standard Size Detached
Confidence in Floor area	Pct Standard Size Semi Detached
Count of Listed Buildings	Pct Tall Flats 6-15 Storeys
	Pct Medium Height Flats 5-6 Storeys
	Pct Tall Terraces 3-4 Storeys
	Pct Very Large Detached
	Pct Very Tall Point Block Flats
	Pct All Unknown Typology

Age	Weather
Pct 1919-1944	HDD
Pct 1945-1959	CDD
Pct 1960-1979	HDD Summer
Pct 1980-1989	CDD Summer
Pct 1990-1999	HDD Winter
Pct Post 1999	CDD Winter
Pct Pre 1919	

The full variable list and metadata are given in the SI table 2026_NEBULA_ariables_list.csv. A more detailed variable list is given in Data Records Section.

**Table 2:** Nebula Variable List Part 2.

Region	Energy
Region	Annual Gas / Electricity
Output Area	Mean Gas / Electricity
LSOA	Median Gas / Electricity
MSOA	Count of Meters (Gas)
Local Authority	Count of Meters (Elec)
**Housing Density**	
Postcode Area	Postcode Housing Density
**Socio-Demographics**	
Ethnicity	Household Size
Economic Activity	Occupation
Household Composition	Central Heating
Tenure	Sex
Occupancy Rating	Deprivation
Deprivation	Highest Qualification
Socioeconomic Classification	Bedroom Count

**Table 3:** NEBULA Data Sources.

Dataset	Frequency	Year	Ref	License
Building Stock	Annual	2022	Verisk^16^	EDU
Temperature	Monthly	2022	Met Office^21^	OG3
UK Regions	Annual	2021	ONS^37^	OG3
Energy	Annual	2022	DESNZ^15^	OG3
Postcode	Quarterly	2022	OS^18^	EDU
UK Census	Decade	2021	ONS^25^	OG3
Urban/Rural classification	Decade	2021	ONS^38,39^	OG3

ONS: Office for National Statistics. DESNZ: Department for Energy Security and Net Zero. OS: Ordnance Survey, © Crown copyright and database rights 2024 Ordnance Survey (AC0000851941). OG3: Open Government v3.0. The UK Open Government Licence is a worldwide, royalty-free, perpetual, non-exclusive licence. EDU: Education licence refers to free access for those in non-commercial research or educational settings.

**Table 4:** Lookup tables and other assorted data sets used in the dataset generation process.

Dataset	Name	Year	Ref	License
ONS UPRN Directory	ONSUD Dec 2022	2022	ONS	OG3
Postcode Centroids	ONSPD Online Latest Centroids	Ongoing	ONS	OG3
Postcode Lookup	PCD to OA to LSOA to MSOA	2021	ONS	OG3

After describing the variable generation, we discuss the filters applied to the data to create a data sample suitable for gas-energy modelling. We restrict to only postcodes which are wholly domestic, excluding mixed-use or commercial postcodes. We apply filters on some of the key building stock variables to exclude outliers and anomalies.

#### Energy

The energy data within NEBULA is drawn from the public records published by the UK Department for Energy Security and Net Zero (DESNZ), downloaded at (Energy URL, Postcode level gas statistics: 2022, Postcode level electricity statistics: 2022) (DESNZ^15^). This data contains annual domestic energy consumption per postcode, with variables including the count of meters per postcode and total, median and mean consumption per postcode, for both gas and electricity. DESNZ provides energy consumption at the postcode level for postcodes with at least five meters, and gas data is regionally adjusted for the temperature to allow for inter-year comparison. Here we use real energy data from 2022.

The gas data is categorised into ‘Domestic’ or ‘Non Domestic’ based on a kWh usage threshold (73,200 kWh). This hard limit means that there will be cases of misclassification, both of small non-domestic users classified as domestic, and cases where multiple properties are served by the same gas meter, which are then classified as Non-domestic. We mitigate this by including only those postcodes which are categorised as wholly domestic based on the building typology, excluding any mixed-use postcodes.

#### Building Stock

The building stock dataset used in this work is the Verisk ‘UK Buildings’ dataset (The GeoInformation Group Ltd^16^), published by the Edina Digimap service (https://digimap.edina.ac.uk/). The Verisk data includes key attributes such as building footprint, height, age and typology for all buildings in the UK. We chose this building stock data over open open-source alternatives due to the greater accuracy of building footprints as noted in Krapf *et al*.^17^. Verisk have confirmed the publication of the NEBULA dataset as acceptable for open source, given that building-level data is not included.

We use several mapping and lookup files to link postcodes and buildings. For the postcode shapefiles, we use the Ordnance Survey (OS) Code-Point® with Polygons (OS^18^, https://www.ordnancesurvey.co.uk/products/code-point-polygons, accessed via Edina Digimap Services https://digimap.edina.ac.uk/) under an Education Licence). To connect the building UPRN to Postcodes, we use the ONS UPRN Directory for December 2022 (ONS^19^, https://www.data.gov.uk/dataset/8e8e5386-df9c-4f78-862a-f17e09f14bba/ons-uprn-directory-december-2022, available under an Open Government v3.0 Licence. We link postcodes to the postcode centroids coordinates from the ONS ONSPD Online Latest Centroids (ONS^20^, https://geoportal.statistics.gov.uk/datasets/ed28dda8b75146288d7d08d56d2290a0_1/explore) available under an Open Government v3.0 Licence.

Before deriving postcode attributes, we implemented cleaning and pre-processing stages. Building heights and ages were cleaned and categorised into discrete groups. Here, height refers to the height of the building, including the roof. While some limited data on roof types is available within Verisk data, the primary attribute provided is roof material rather than shape. Various errors exist within the dataset. We focused on reducing the errors in floor count and height based on thresholds derived from average building parameters. A key variable is the total building floor area within the postcode. We first derive gross external area (GEA) and subsequently, gross internal area (GIA).

Building floor area can be derived either using the storey count variable or the building height variable. To make best use of the data available and enable a confidence assessment of the variable, three methods are used to derived GEA: directly from the raw storey count (GEA_Raw_), a validated storey count method (GEA_Val_) and directly from the height variable (GEA_Height_).

For GEA_Raw_, the derivation is

1 $${{\rm{GEA}}}_{{\rm{Raw}}}={{\rm{FC}}}_{Raw}\times A$$

Where FC_*Raw*_ is the raw storey count and *A* is the area of the building footprint.

For GEA_Val_, the storey count is validated before the area calculation. We first derive the average floor height *F*_*H*_:

2 $${F}_{H}=\frac{H}{{{\rm{FC}}}_{Raw}}$$

where *H* is building height and FC_*Raw*_ is building floor count. We calculate the minimum edge of the building footprint, defining it as the width *W*_*M*_.

We validate the height and floor count for each building using the following algorithms:

3 $$\,{\rm{if}}\,\,H\ge 3\cdot {W}_{M}\,\,{\rm{OR}}\,(H < 2\,{\rm{AND}}\,{\rm{numeric}}\,({{\rm{FC}}}_{raw}))\ \Rightarrow \ H=\,{\rm{NULL}}$$

4 $$\,{\rm{if}}\,\,({{\rm{FC}}}_{Raw} < ={{\rm{thresh}}}_{min}\,\,{\rm{OR}}\,\,{{\rm{FC}}}_{Raw} > {{\rm{thresh}}}_{max})\,\,{\rm{AND}}\,H < 3\cdot {W}_{M}\ \Rightarrow \ {{\rm{FC}}}_{Raw}={\rm{NULL}}$$

Height is set to invalid if height is greater than three times the *W*_*M*_ or if the height is less than 2m, and there is a valid floor count present. Floor count is set to invalid if outside the thresholds for average floor height and if height is less than 3x *W*_*M*_. Missing or null values for *H* and *F* are then filled using local averages from within the postcode: FC_*val*_.

The thresholds for average floor height (thresh_*min*_ and thresh_*max*_) are set per typology and are extracted from global results of the national dataset. For each typology the mean and standard deviation for average floor height is calculated, and the thresholds are set as mean  ±2  × std. A lower-end cap is enforced at 2m based on physical ceiling height limits. These thresholds are smaller for flat typologies and larger for single-family home typologies. The typology floor height thresholds are given in Table 4 in the SI. The thresholds range from 2 m to 6.9 m. Setting thresholds per-typology enables better error detection. We validate these thresholds against the UK EPC data. The extracted ranges from EPC for internal floor height are 2.1 m–3.9 m for the IQR across regions (details given in Section 2 of the SI). To convert these to external height, we apply a scaling factor of 1.6, leading to an upper threshold of 4.6 m. Given the presence of roofs of various heights and also taller older buildings, the upper threshold of 6.9 m is acceptable.

Hence:

5 $${{\rm{GEA}}}_{{\rm{Val}}}={{\rm{FC}}}_{{\rm{Val}}}\times A$$

Finally, GEA_Height_ is calculated using a lookup to the global average floor count for heights, per age and typology. The global average floor counts for each building and age combination are calculated across the whole national dataset, from all the buildings in the UK Buildings dataset. These values are given in the SI. For every building, a floor count can hence be calculated based on global averages derived from its height (FC_Height_).

6 $${{\rm{GEA}}}_{{\rm{Height}}}={{\rm{FC}}}_{{\rm{Height}}}\times {F}_{A}$$

An uncertainty spread metric (U) is constructed based on the spread across the three metrics:

7 $$U=\frac{({\mathrm{GEA}}_{\max }-{\mathrm{GEA}}_{\min })}{{\mathrm{GEA}}_{\mathrm{mean}}}\times 100$$

where GEA_min_, GEA_mean_ and GEA_max_ are calculated on the group [GEA_Height_, GEA_Raw_, GEA_Val_].

A ‘confidence’ metric (C) for floor area is then derived by bucketing U. The thresholds for the confidence metric area are dervied on the quartiles of the national dataset:

- High Confidence: *x* < 0.5% difference
- Medium Confidence: 0.5 < *x* ≤ 6% difference
- Low Confidence: 6 < *x* ≤ 18% difference
- Very Low Confidence:  > 18% difference

The uncertainty metric U and the confidence metric C identify buildings where GEA estimates diverge significantly. In cases where height and floor count data align closely, a small spread leads to a high confidence, whereas discrepancies between them indicate potential data quality issues and lead to a low confidence.

We then derive an estimate of gross internal area (GIA), from GEA, using EPC floor area assessments. We match Unique Property Reference Numbers (UPRNs) from Verisk building stock to EPC data and calculate the ratio of GEA to Floor Area for any non-flat typologies. We calculate the global means for each combination of typology and age for this scaling factor (filtering to only cases with at least 1000 samples) and use it to scale the GEA for each building based on its typology and age. For cases where the specific typology and age combination does not have a sample scaling factor, we use the global mean scaling factor across all matched buildings, and failing that, the overall average scaling factor of 0.6. For flats, it is not possible to reliably calculate the number of flats within each block at scale, and so the standard scaling factor is used. Further details of the GEA scaling factor are given in the SI. Three GIA metric are derived, based on scaling the three GEA metrics: GIA_*Height*_, GIA_Raw_, GIA_Val_.

**Fig. 1:**
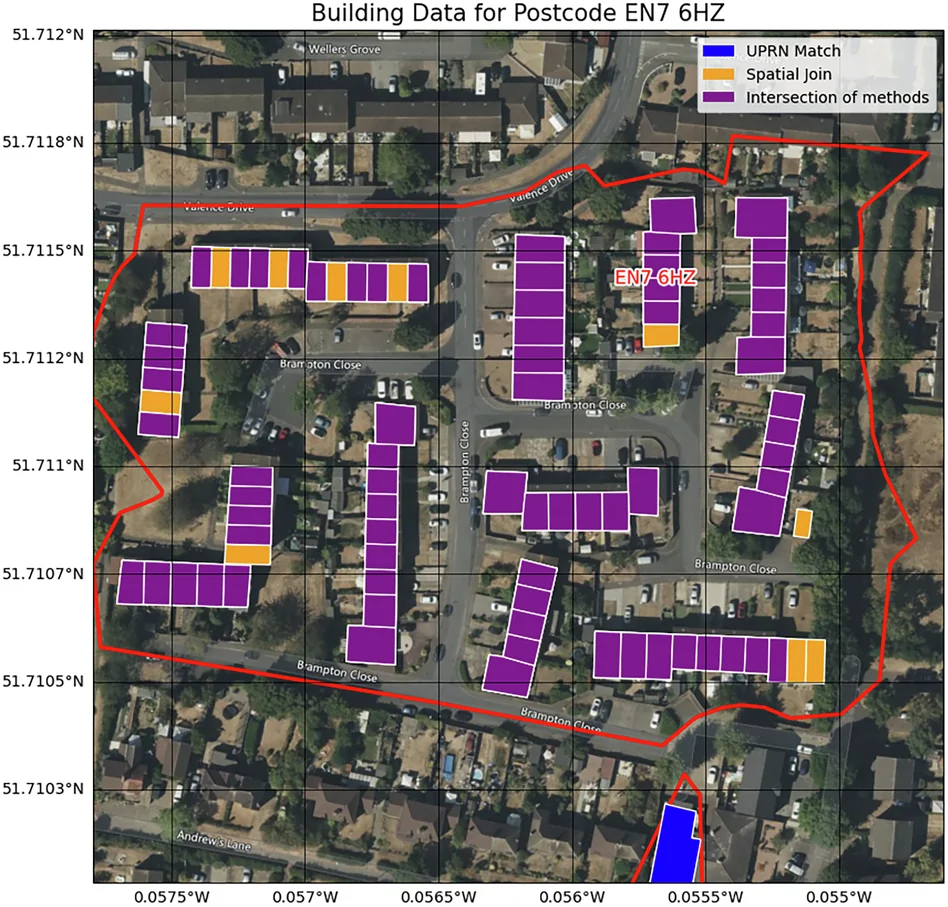
Overhead satellite image showing an example of the dual method for matching buildings to postcode. Visualisation code adapted from Lipson^35^. Satellite image downloaded from^36^. Footprints via Digital Map Data © Verisk (The GeoInformation Group Ltd.)^16^.

After dataset pre-processing, all buildings within *PC* are identified, using a spatial join between building footprints and postcode polygons and a UPRN to Postcode mapping. This identifies both dwellings and outbuildings (structures with no UPRNs). Figure 1 shows an example of this matching. The cleaned building stock for *PC* is then aggregated into small neighbourhood-level variables. Variables are either count, total sum or percentage, e.g. count of residential buildings, total GEA for residential buildings and percentage of residential buildings in a postcode.

#### Climate

We include a postcode-level climate variable calculated from the 2022 monthly temperature data from HADUK an open-source dataset from the UK Met Office (Met Office^21^, https://www.metoffice.gov.uk/research/climate/maps-and-data/data/haduk-grid/haduk-grid. The dataset is at 1km resolution, and for each postcode, we identify the nearest temperature sensor to calculate temperature variables.

Heating and Cooling Degree days are calculated from monthly Temperature data using Equations (8) and (9). The closest temperature sensor to the postcode is identified, and monthly values for HDD and CDD are calculated using the set points of 18 and 15.5.

8 $$\,{\rm{HDD}}=Min(15.5-{\rm{Temp}},0)$$

9 $$\,{\rm{CDD}}=Min({\rm{Temp}}\,-18,0)$$

Pre-processing of temperature data includes spatial interpolation of nulls in the x and y direction, with a limit of 10 km (limit of 10 neighbour fills). HDD and CDD are calculated for summer, winter, and annually for 2022, using six-month windows, where 1 = January.

10 $${\rm{summer}}\,{\rm{months}}=[4,5,6,7,8,9]$$

11 $${\rm{winter}}\,{\rm{months}}=[1,2,3,10,11,12]$$

#### Area and Housing Density

We include variables on both the area of the postcode and the housing density. We use the postcode shapefiles mentioned above to calculate the area of the postcode *P*_*A*_, using the Python library GeoPandas^22^. Postcode density *P*_*d*_ is calculated:

12 $${P}_{d}=\frac{{\sum }_{i}\,{A}_{i}}{{P}_{A}}$$

where *A*_*i*_ is the building footprint area. We also include the 2021 Urban/Rural classification data here from the UK Government, as tagged in Table 3.

#### Regions

We match *PC* to Output Area (OA), and subsequent higher geographic census regions (Lower Super Output Area (LSOA), Middle Super Output Area (MSOA) and Local Authority (LA)). administrative regions using the ONS Postcode Lookup for the 2021 census geographies (ONS^23,24^, https://geoportal.statistics.gov.uk/datasets/e7c49b62898a417192a336aca17e3a3f/about).

#### Socio-Demographics

We uses the 2021 census for England and Wales to calculate socio-demographic data per *PC* (ONS^25^, https://www.ons.gov.uk/census/planningforcensus2021/ukcensusdata). The raw census data (e.g. count of households with property X) is transformed into percentage attributes at the OA level and then matched to *PC*, the aforementioned ONS. The 13 themes of demographics are given in Table 2. The census themes were chosen to cover a broad range of socio-demographic factors and include highly predictive attributes identified in prior works. The total number of demographic attributes is 125.

#### Energy Use Intensity

Energy Use Intensity (EUI) is calculated as annual energy consumption per gross domestic internal floor area (GIA). This is a key metric for assessing energy consumption normalised by building size. We calculate the total heated floor area per PC using the GIA of all domestic buildings (excluding outbuildings), using GIA_Raw_:

13 $$\,{\rm{EUI}}=\frac{E}{{\sum }_{{\rm{PC}}}\,{{\rm{GIA}}}_{{\rm{Raw}}}}$$

where *E* is the total annual domestic energy consumption (gas or electric) and GIA_Raw_ is the GEA_Raw_ scaled by the associated scaling factor, summed over the postcode.

### Final Dataset

After attribute generation, several additional filters were applied to create a clean dataset.

#### Residential Filter

We filter only to include postcodes that are identified as 100% residential. This is to match the postcode-level gas and electricity data; non-domestic energy data is not available at the postcode level.

#### Household filter

We filter based on the alignment of the number of gas meters and the count of UPRNs in a postcode. UPRNs refer to addresses and work as a proxy for the count of households. We filter out any postcodes where the difference between the count of UPRNs and the count of gas meters was greater than 40%. This excludes any areas with a high proportion of properties not connected to the gas grid (‘non-gas’ properties). Given our stated aims of a dataset to support energy modelling of gas consumption for policy-makers targeting net zero, this enables us to investigate postcode-level EUI with confidence. Users of this data set should bear this limitation in mind, and future iterations of the data set should expand to include this element.

#### EUI thresholds

Whilst energy analyses typically exclude based on total energy use, here we follow^12^ and filter based on annual EUI thresholds (kWh/m^2^). A gas EUI filter of 5 < EUI  < 650 kWh/m^2^ and an electricity filter of 5 < EUI  < 150 kWh/m^2^ are applied.

#### Other thresholds

Thresholds were applied to the count of buildings and total building GEA of 1–100 and 50–20,000 m^2^ respectively. We also exclude postcodes with a percentage of unknown typologies  > 25, and postcodes with average gas  < 65,000 kWh/yr or total gas  < 1,000,000 kWh/yr. We also cap the postcode area at 2.5e+05 *m*^2^.

**Fig. 2:**
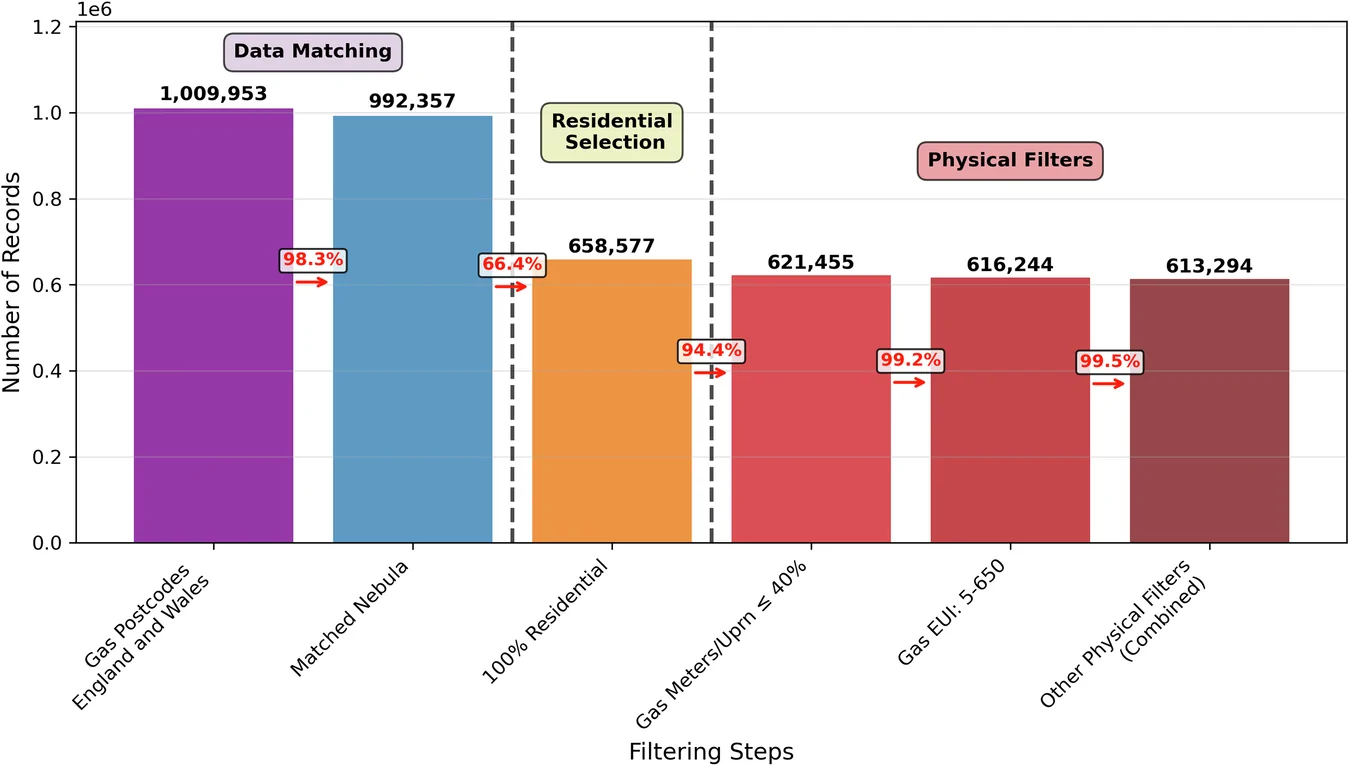
Infographic showing the stages of the NEBULA data pipeline. Each processing step is shown along with the count of postcodes for the NEBULA postcode matching, residential selection and physical filters.

**Fig. 3:**
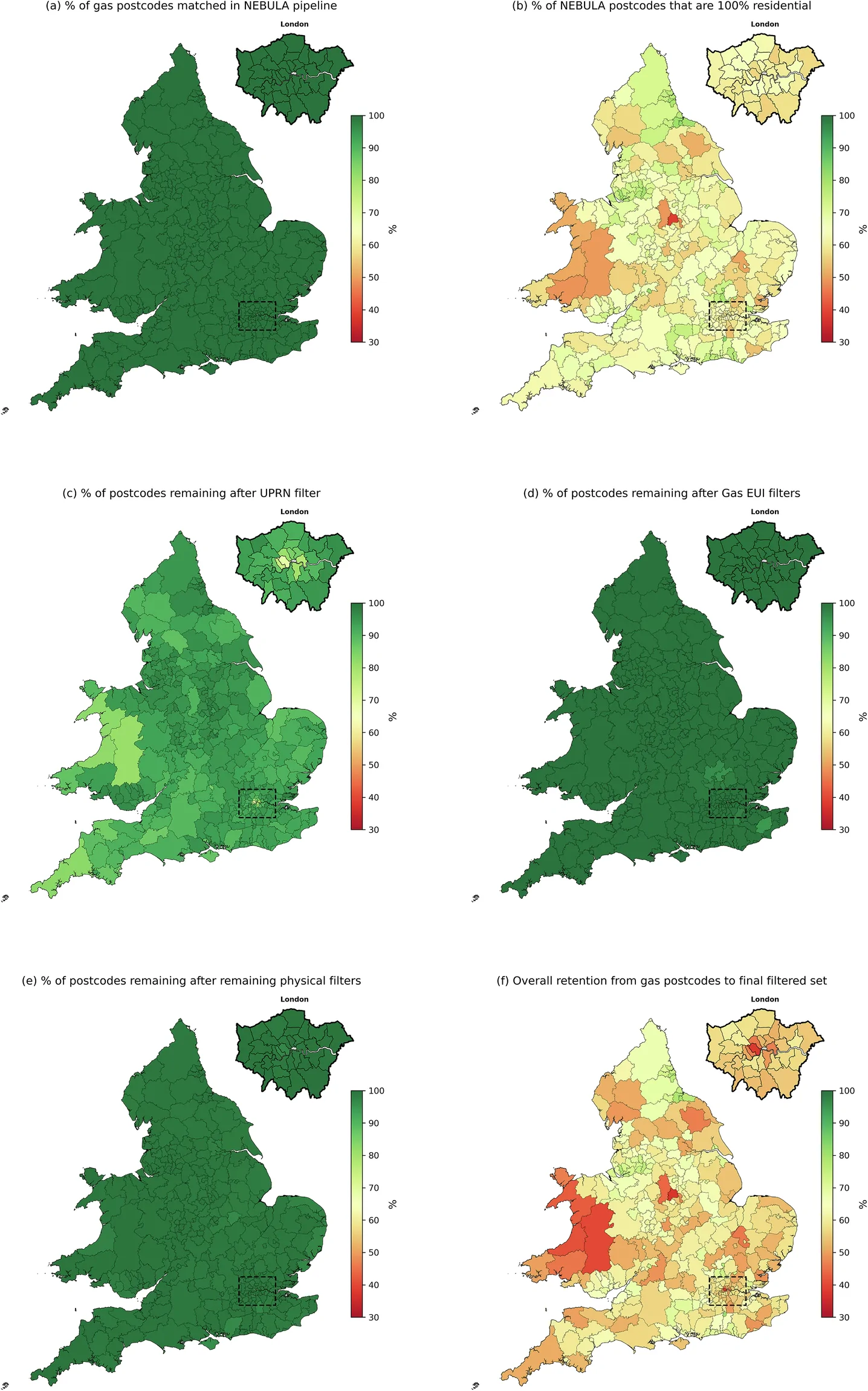
Spatial analysis of residential gas distribution and postcodes processing during the NEBULA data pipeline, shown by Local Authority District (LAD). Insets show Greater London. Excluding City of London LAD.

The final NEBULA dataset contains 613,290 samples. A visual representation of the processing steps and dataset sizes is given in Fig. 2, and the summary statistics for the dataset are given in Table 5. Spatial distributions of the pipeline are given in Fig. 3. 98% of the postcodes with gas data in 2022 for England and Wales are identified, matched and processed in the NEBULA pipeline. 66% of these postcodes pass the wholly domestic filter, based on building typology. Of these, 94.4% pass the UPRN filter, a subsequent 99.2% pass the EUI filters, and 99.5% pass the rest of the filters. In total, the physical filters remove 7% of the data, with the final data sample of 613 k representing 93.1% of the residential postcodes with gas data.

**Table 5:** Summary of final dataset, with mean, 25 th, 50 th and 75 th percentile.

Metric	PC Area (m^2^)	COB	GEA (m^2^)	GIA (m^2^)	Total (kWh/yr)		Num. meters		EUI (kWh/yr/m^2^)	
					*Gas*	*Elec*	*Gas*	*Elec*	*Gas*	*Elec*
mean	12,051	20	2,854	1,713	230,518	63,802	21	22	139	39
25%	4,316	9	1,467	869	114,491	31,743	11	11	113	30
50%	7,503	17	2,421	1,451	193,625	53,886	18	18	135	37
75%	12,938	28	3,793	2,299	311,155	86,307	29	29	160	46

PC Area: postcode area. COB = count of buildings. Fl.area = total residential building floor area.

Examining the spatial distribution of the filtering process in Fig. 3 shows that the pipeline does not have an equal spatial distribution. The plots (a)-(f) show the sequential filtering stages, where each reported percentage represents the proportion of postcodes excluded relative to those remaining from the preceding step. Importantly, these filtering effects are cumulative: for instance, the 70% exclusion rate following the gas EUI filter applies only to postcodes that had already survived the UPRN filter, not to the original dataset. The statistics are aggregated to the Local Authority (LAD) for visualisation purposes. While there is a strong, mostly uniform match from the gas postcodes to the NEBULA postcodes, as shown in Fig. 3(a), the residential filter has unequal spatial implications: Fig. 3(b)showing the percentage of wholly residential postcodes highlights several LADs in East Midlands and Wales as being particularly low in wholly residential postcodes. Amber Valley, Broxtowe and Derbyshire Dales in the East Midlands, and Carmarthenshire, Ceredigion, and Powys in Wales are among the LADs with the lowest percentage of wholly residential postcodes, ranging between 38–50%. Figure 3(c) shows the percentage of postcodes remaining after the gas meter count comparison to the UPRN filter, which also shows an interesting spatial trend. Parts of London (Westminster, Kensington and Chelsea, Tower Hamlets, Southwark and Camden), and Wales (Powys, Ceredigion and Gwynedd) have the highest dropout rate based on gas meter count to UPRN count, with a pass rate between 68 and 82%. The gas EUI filter and other physical filters, as shown in Fig. 3(d),(e), remove very few additional postcodes. Examining the overall retention rate in 3(f), the residential filter accounts for the majority of exclusions. The summary tables for the filtering stages are given in the SI (Table LAD_filtering_nebula_pipeline.csv). The exclusion of mixed-use and commerical postcodes is an important limitation for the user to bear in mind when using this data.

## Data Records

The dataset is deposited in the University of Cambridge APOLLO repository. The dataset takes the form of the following files:

- Dataset files:
  - Dataset 1: NEBULA Dataset Domestic Filtered. Shape: [613290, 232] NEBULA_englandwales_domestic_filtered.csv
  - Dataset 2: NEBULA Dataset Domestic Unfiltered. Shape: [658577, 232]. NEBULA_englandwales_domestic_unfiltered.csv
  - Variables list: 2026_NEBULA_Variables.csv
  - Variables Read me: 2026_NEBULA_Varaibles_README
- Supplementary Information Folder (SI)
  - Supplementary Information PDF SI.pdf
  - Typology / Age Global Average table: Global_average_floor_count_bucketed.csv
  - NEBULA Filtering Pipeline LAD results: LAD_filtering_nebula_pipeline.csv

The metadata given for the variables in the dataset tables (NEBULA_englandwales_domestic_unfiltered.csv and NEBULA_englandwales_domestic_filtered.csv) is shown in Table 6. These metadata variables (excluding the notes) are provided for all variables. The full list of variables and metadata is given in the table provided with the data download 2026_NEBULA_Variables.csv^26^.

**Table 6:** Dataset variables metadata columns.

Column Name	Description
Data Type	e.g. string, integer, boolean
Category	Geography, Building Stats, Climate, Energy, Building Age
	Building Typology, Socio-demogs
Direct/Derived	Direct variables comes directly from noted data sources.
	Derived are calculated using methodology set out in the paper
Source	Main data source relating to the variable
Description	Variable definition
Notes	Notes on usage
Units	Units where applicable

As discussed in the Building Stock Section, there are three versions for each of the building floor area metrics, reflecting different derivations. The default recommended are the residential_GEA and the residential GIA for the GEA and GIA metrics, representing GEA_Raw_ and GIA_Raw_ respectively.

## Technical Validation

### Morris Analysis

The pre-processing stages detailed in the methodology have corrected various errors in the underlying building stock data. Given the aforementioned prior work within the literature identifying square meters of living space as a highly influential variable influencing energy consumption, we run a sensitivity analysis on our derived variables relating to GEA (Raw, Val, Height). Our approach, informed by Menberg *et al*.^27^, employed Morris’ method for parameter screening, which systematically explores the parameter space by applying one-at-a-time variations to input parameters^28^. This method was chosen for its computational efficiency, given the substantial run time of the data generation model, and the ability to identify the variation caused by each input parameter. For each of the 10 regions in England and Wales, we tested a sample of 5000 postcodes, stratified on the number of buildings per postcode to ensure representativeness. The analysis involved applying perturbations of  ±10% to height and floor area, sampling perturbation multipliers from 0.9 to 1.1 for each input.

For a set of k input parameters, Morris’ method generates a sequence of k+1 points, each differing in one coordinate from the preceding one, forming a trajectory, where each point represents a model run. The magnitude of variation in model output due to a pre-defined variation of parameter i is termed the elementary effect (EE), calculated as^27,28^:

14 $${EE}_{i}=\frac{Y(X+{e}_{i}{\Delta }_{i})-Y(X)}{{\Delta }_{i}}$$

where Y is the model output, X is the parameter vector, *e*_*i*_ is a vector of zeroes except for the i-th parameter that equals 1 and represents an incremental change in parameter i, and *δ*_*i*_ is the predetermined variation. EE has the same units as the model outcome Y, which for our analysis is *m*^2^.

For each postcode, we run 15 Morris trajectories, examining the absolute mean (${\mu }_{i}^{\ast }$) and standard deviation (*σ*) of the elementary effects:

15 $${\mu }_{i}^{\ast }\,=0.5\,\mathop{\sum }\limits_{t=1}^{r}| {EE}_{it}| $$

16 $$\sigma =\sqrt{\frac{1}{(r-1)}\mathop{\sum }\limits_{t=1}^{r}{({EE}_{it}-{\mu }_{i})}^{2}}$$

where r is the number of trajectories.

Menberg *et al*.^27^ noted that the Morris method could be unstable regarding parameter ranking in the context of building energy modelling. Still, that instability was mitigated by using the absolute median and multiple runs. Given the large number of postcodes being analysed relative to the trajectories, we calculate both the mean and median across the ${\mu }_{i}^{\ast }$ and *σ* across the set of postcodes. Given the high computational cost per postcode and the range of postcodes assessed, this method efficiently compromises accuracy and computational feasibility.

**Fig. 4:**
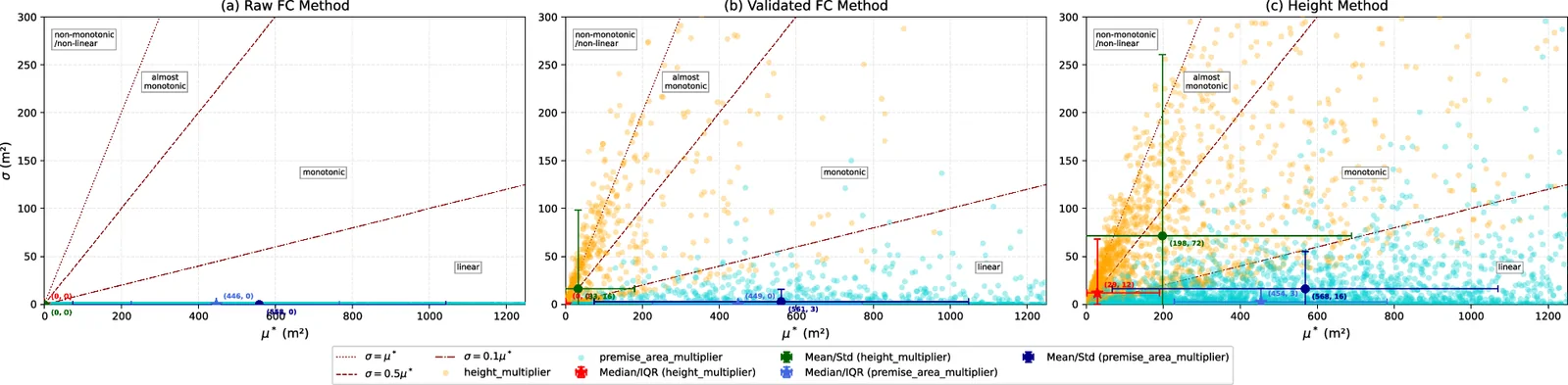
Morris sensitivity analysis (*σ* vs μ^*^) across all regions (53,655 postcodes) for the three GEA metrics: Raw FC, Val FC and Height method. Median and IQR ranges are plotted, along with mean and standard deviations. The Premise area multiplier exhibits high μ^*^ with low *σ* across all three estimates, indicating a strong, linear influence. Reference lines delineate regions of linearity (*σ* = 0.1μ^*^), monotonicity (*σ* = 0.5μ^*^), and non-linearity (*σ* = μ^*^).

The results from evaluating 53,655 (at least 5 k per region) postcodes with the Morris method with 15 trajectories are given in Fig. 4, which contains the visualisation across the runs for the three GEA variables. Table 7 contains the values of the Morris parameters. Following the classification scheme proposed by Garcia Sanchez *et al*.^29^ and applied by Menberg *et al*.^27^, we analysed the ratio of μ^*^ to *σ* to characterise input parameters in terms of (non-)linearity and (non-)monotony. The three GEA estimates show the same pattern of behaviour for premise area, identifying it as the more influential parameter with higher elementary effects and consequently a greater impact on Y. The median results for premise area are lower than the mean, due to the influence of outlier postcodes, a phenomenon also observed by Menberg *et al*.^27^. Notably, the premises area consistently falls within the linear regime, indicating a direct linear relationship between variations in the premises area and GEA per postcode, an expected outcome given the model’s algorithmic structure. In contrast, the three methods show distinct behaviour as related to height, as expected given their different derivations. The Height method shows a substantial impact from the height multiplier, with a non-zero median and mean value (median 29 m^2^ vs. mean 198 m^2^ for *μ*), along with significantly higher outlier impact (higher *σ*). The validated FC method shows a reduced primary impact: the median postcode shows no impact from the height multiplier, while the mean shows a reduced impact (33 m^2^) reflecting the fact that the validation only catches those buildings outside of certain floor height thresholds, and so only a subset are changed by perturbing the height. The Raw Floor Count method shows no impact from the height multiplier, reflecting the lack of involvement of height in the methodology. For both the Height method and Validated Floor Count method, the height multiplier demonstrates monotonic to almost-monotonic behaviour, characterised by a substantially lower μ^*^ but a much higher *σ* than the area multiplier. For the Validated Floor Count method, this reflects the complexity in the relationship from height to floor count and the thresholds, and for the Height method, the relationship between the perturbed height and the global average floor counts.

**Table 7:** Morris parameter results.

Metric	Variable	μ^*^					*σ*				
		mean	std	median	Q25	Q75	mean	std	median	Q25	Q75
Floor Count Raw	Height	0	0	0	0	0	0	0	0	0	0
	Premise Area	558	485	446	225	767	0	0	0	0	0
Floor Count Val	Height	33	147	0	0	0	16	82	0	0	0
	Premise Area	561	486	449	227	771	3	13	0	0	0
Height	Height	198	490	29	2	191	72	189	12	1	68
	Premise Area	568	501	454	229	781	16	39	3	0	16

For the Validated Floor Count method, a 10% change in premises area equals a mean 561 ± 3 m^2^ (μ^*^  ±  *σ*) absolute change in Y (total postcode floor area), whilst a 10% change in height equals a mean 33  ± 16 m^2^ absolute change. Set against the mean Y of 2,856^2^ (refer to Table 5), the potential variation due to 10% variation in premises area is 19.6 ± 0.1%, and in height is 1.15  ± 0.5%.

The Morris results for the three GEA metrics are provided both to validate the metrics construction and to highlight the differences between them. If notable local issues in either height (from the UK LiDAR scan) or storey counts are known, a different metric can be chosen by the end user.

**Fig. 5:**
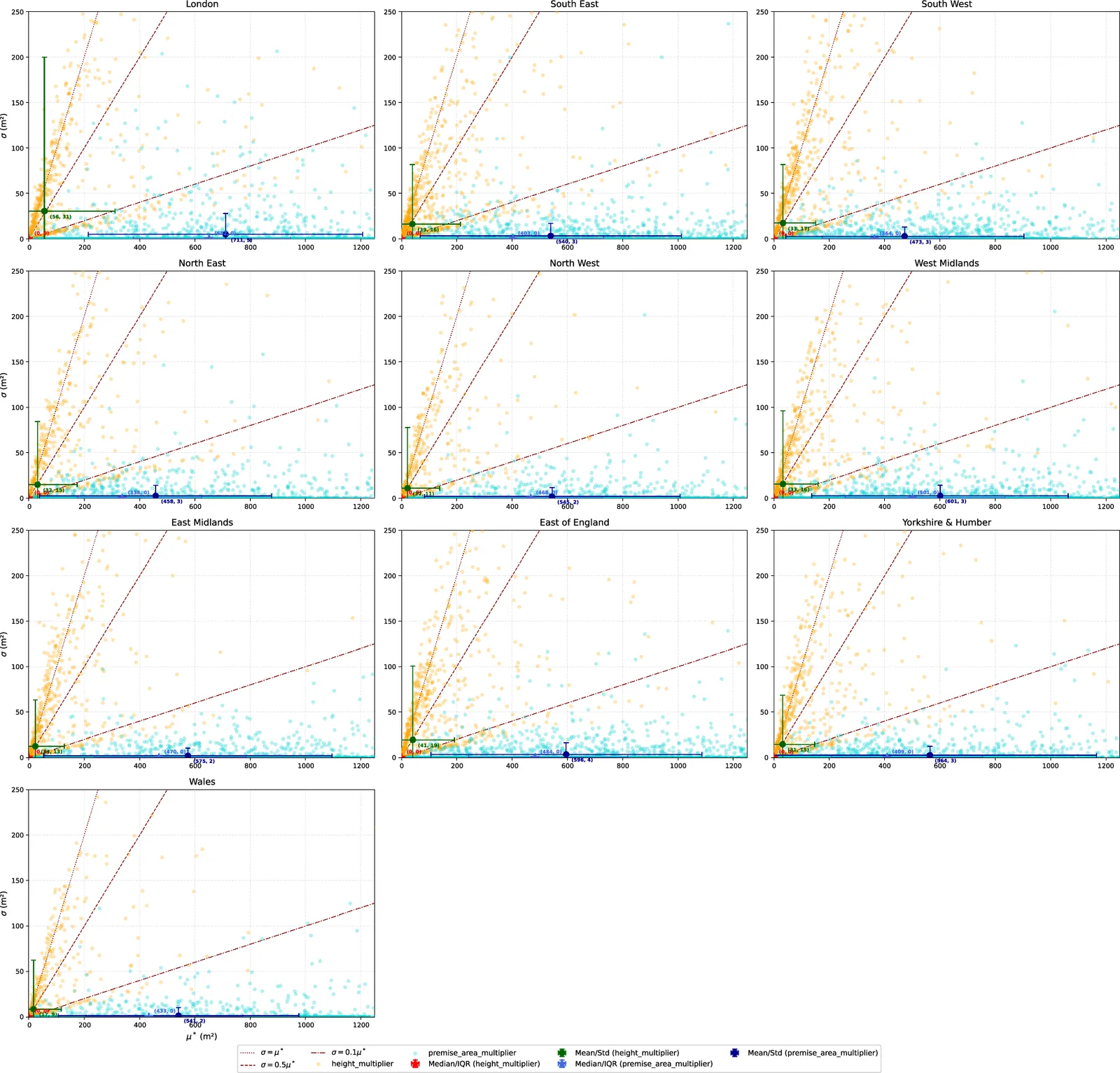
Per-region Morris sensitivity analysis (*σ* vs μ^*^) for the building floor area metric (GEA_Val_, Floor Count Val method) estimate across all 10 regions. Premise area consistently dominates with near-linear behaviour, while height sensitivity varies regionally, with London showing the greatest influence.

**Fig. 6:**
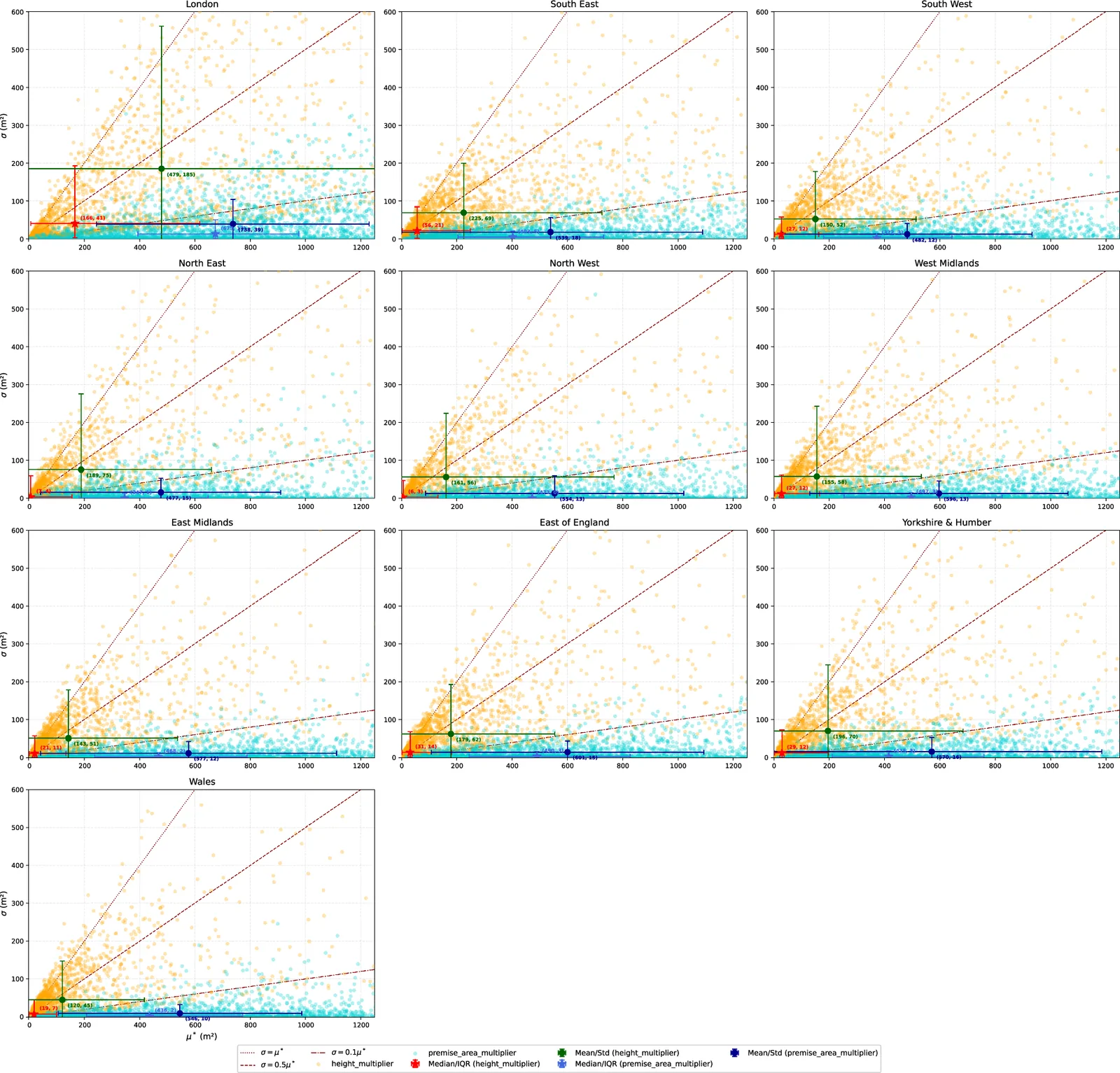
Per-region Morris sensitivity analysis (*σ* vs μ^*^) for the building floor area metric (GEA_Height_, Height and Global Storey Count method) estimate across all 10 regions. Premise area consistently dominates with near-linear behaviour, while height perturbation shows greater impact, varies regionally, with London showing the greatest variation.

The results broken down by region (given in Fig. 5 for GEA_Val_ and Fig. 6 for GEA_Height_) indicate a range of regional performances. In Fig. 5, all regions show consistent results for floor-area perturbations, with a linear influence. For the height perturbation, while all regions have relatively equal performance in the median results, London shows a greater range in the mean influence of the height perturbation, hence a greater number of outlier postcodes. The standard deviation in the London results are significantly higher than those in other regions. This is due to more postcodes having buildings within 10% range of the floor height thresholds per typology, and so being adjusted after validation. In Fig. 6, a similar performance is seen where premise area dominates in the linear regime. Height perturbations have a greater impact here, as expected given the metric. Regional variation highlights London as having both a higher impact from height perturbations in the mean and median, and a higher standard deviation than other regions.

The Validated Floor Count methodology, by construction, uses thresholds on the acceptable floor heights derived per typology (used to exclude those deemed physically unlikely). Investigations revealed that changing these thresholds affects regional performance and should not be taken as indicating an underlying physical pattern in London beyond a greater presence of buildings near the height thresholds, which trigger validation more often than those in the lower strata of the distribution. Similarly, the Height methodology, which includes using the global average storey count per height, will naturally struggle where buildings are at the extremes of their typologies. Given the method of deriving global storey count for each typology and age, the variation for London likely reflects instability in the height-to-storey heuristic.

Given these regional variations, we recommend using the metrics GEA_RAW_ and GIA_RAW_ as the defaults for analysis (hence their use in deriving the EUI metrics). The Height and Val metrics are useful for identifying places where height and storey count diverge. These are particularly useful when working within a region. They are also provided for completeness, given their use in the GEA metric agreement U (Eqn. 7). Cross-regional comparisons of the metric U should note the limitations of the methodology in London.

### Regional Performance

The Morris analysis indicated that London has postcodes that trigger the floor count validation in greater numbers than other regions. Prior work using an earlier version of this dataset (Colverd *et al*.^13^) also indicated London as an outlier region in terms of energy consumption. Given the desired use of this dataset for energy modelling, further investigation into the regional variation is included.

**Fig. 7:**
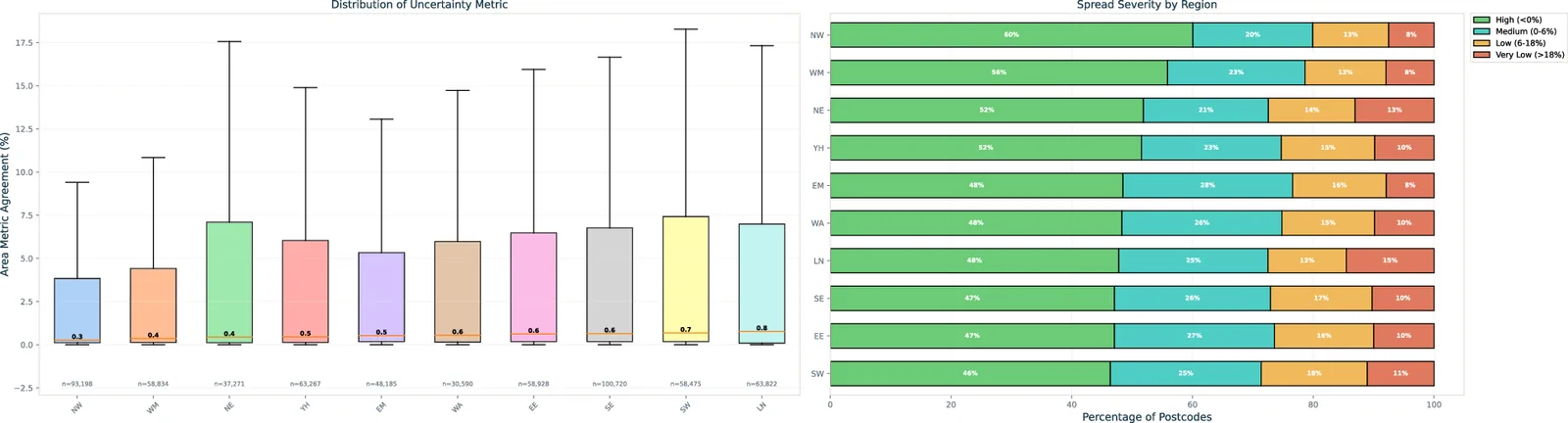
Regional analysis of the agreement of the three building area metrics (GEA_Raw_, GEA_Val_, GEA_Height_), using the metric agreement U and the bucketed metric C.

The regional breakdown of the uncertainty metric U and confidence floor area metric C (as defined in the Building Stock section above) are shown in Fig. 7. London has a similar proportion of High and Medium confidence postcodes to the other regions (Fig. 7(a)), and while it does have a higher proportion of ‘Very Low’ confidence areas (15% vs. group mean of 10.0%), it is not a significant outlier. The box plot distributions of the GEA agreement metric U (Fig. 7(b)) confirm this, with the North East and South West both having similar distributions of metric agreement across the GEA metrics.

**Fig. 8:**
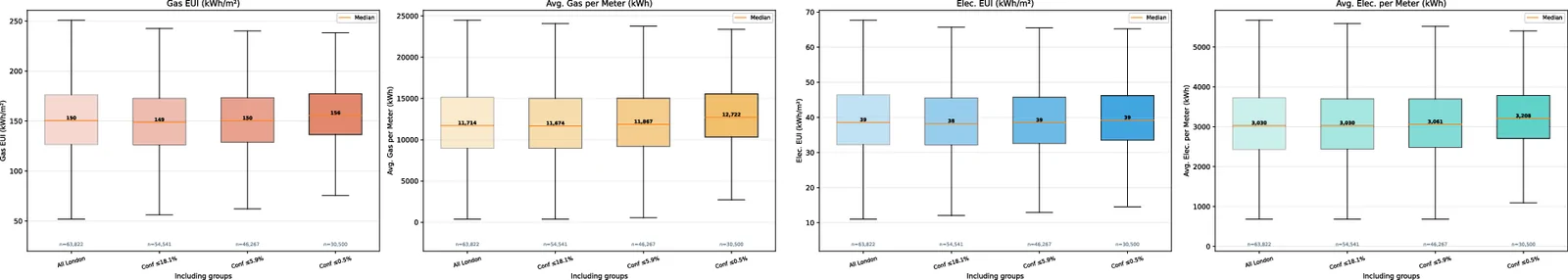
Box plot analysis of the sensitivity of energy metrics (average energy per meter and energy use intensity) in London to the uncertainty in the area metrics, using metric U.

To validate whether these lower confidence regions impact the energy distributions, four metrics are investigated to see the impact of filtering on higher confidence postcodes. Gas EUI, Electricity EUI, both derived using the GIA_raw_, and average gas/electricity per meter, directly taken from the subnational consumption data, are investigated. The filtering for each confidence level for postcodes in London is given in Fig. 8. While excluding the ‘Very Low’ confidence postcodes reduces the spread of the error bars on the box plot, the median values are not changed significantly. For all metrics, the change in spread and median when excluding all except the ‘High’ confidence areas is the greatest. This marginally increases the median Gas EUI (150 kWh/m^2^ to 156 kWh/m^2^), the Avg. Gas (11,714 kWh to 12,722 kWh) and Avg. Electricity (3,030 kWh to 3,208 kWh), while Electricity EUI stays consistent (39 kWh/m^2^). These results indicate no significant change in energy consumption trends when excluding the low-confidence floor areas, validating their inclusion in the final dataset. In addition, these results indicate that no bias of energy consumption is introduced due to the uncertainty in the floor area metrics.

**Fig. 9:**
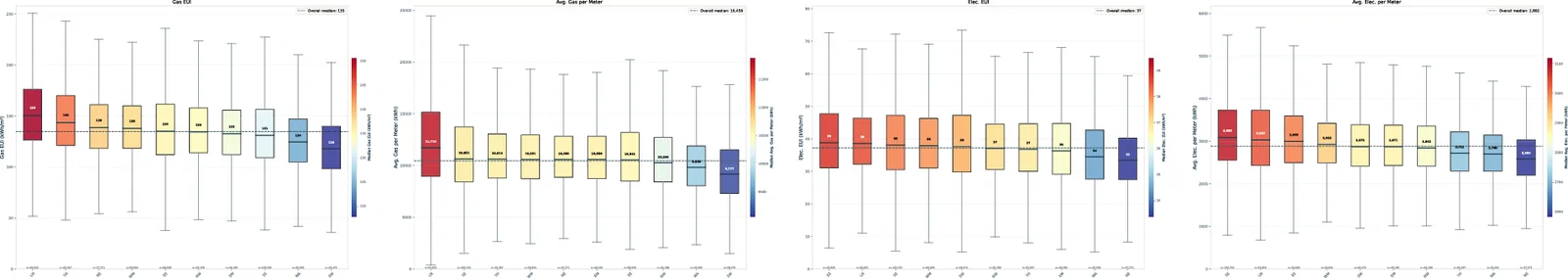
Regional distributions of average energy per meter and energy EUI, for gas and electricity.

**Fig. 10:**
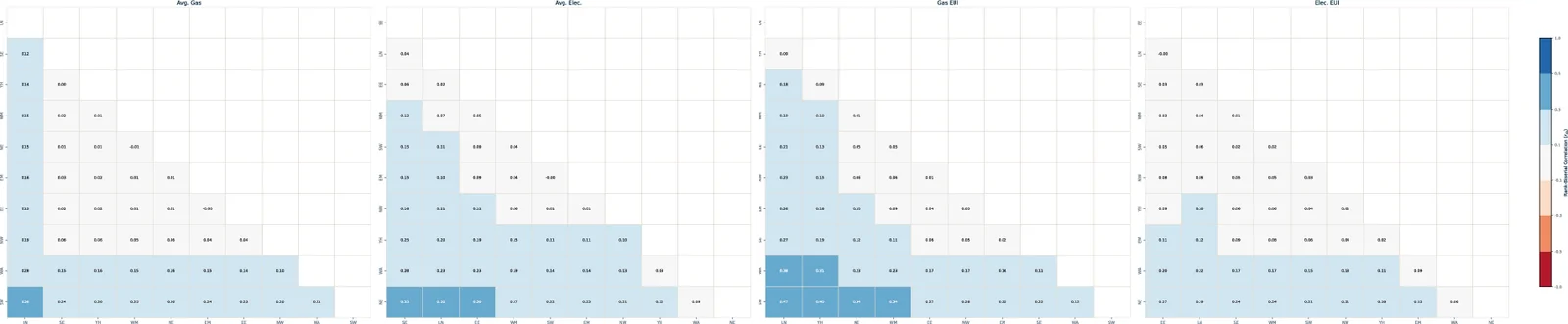
Effect sizes between regions. All regions ordered by median gas EUI. A positive value in the effect size indicates the x-axis region is higher, colour coded by the categorical effect size thresholds: large ≥ 0.5, medium ≥ 0.3 and small ≥ 0.1.

The regional distribution for these four energy metrics are shown in Fig. 9. Each plot is ordered by the group medians of the dataset. Given the wide distribution of values indicating potential significant overlap in the regional performance, the statistical significance of the differences between regions is tested pairwise using the Mann-Whitney U statistic (selected due to the non-normal distribution of energy data). Given the large sample size, the effect size is computed using Cliffs Delta, chosen due to its robustness with non-normal distributions^30^. The Bonferroni correction is applicable given the large number of tests conducted. The standard ranges of large ≥0.5, medium ≥0.3 and small ≥0.1, are applied. The results of the significant test effect size are given in Fig. 10. Positive values indicate that the column is larger than the row.

- **Gas EUI** the top two consuming regions (London, median 150 kWh/m^2^, Yorkshire 144 kWh/m^2^) have a medium effect size compared to the two bottom consuming regions (South West 118 kWh/m^2^ and Wales 124 kWh/m^2^) and a small effect against the remaining six regions, except for between Yorkshire and the North East. The lowest region (South West 118 kWh/m^2^) also has medium effects against the next top 2 regions (North East 139 kWh/m^2^, West Midlands 138 kWh/m^2^), and small effects against the other regions. Based on the effect sizes and distributions, three groupings are suggested. High consumers: London and Yorkshire. Low consumers: Wales and the South West. Middle groupings: the remaining regions.
- **Electricity EUI** The lowest two regions (North East median 33 kWh/m^2^ and Wales 34 kWh/m^2^) show small effects compared to all regions except each other, and except for Wales and East Midlands. The third lowest (East Midlands 36 kWh/m^2^) has small effects compared to London and East of England. London also has a small effect compared to Yorkshire. Based on the effect sizes, the only notable group is the low-performing group of Wales and the North East, with the remaining regions overlapping in distribution, such as to reduce any significant effects between them.
- **Average Gas per Meter** the top region (London, median 11,714 kWh) has a medium effect compared to the lowest region (South West, median 9,177 kWh), and small effects against the remaining 8 regions. The bottom two regions (South West 9,177 kWh and Wales 9,650 kWh) have low effects compared to all other regions except for each other. Based on the effect sizes, the top performing group is London and the lowest performing group is South West and Wales, with all other forming a middle consumption group.
- **Average Electricity per Meter** The top three regions (South East median 3,086 kWh, London 3,030 kWh, East of England 2,995 kWh) are identified as the high consumption group, with medium effect against the lowest region (North East 2,584 kWh) and small effects against the majority of other regions except each other. The lowest three regions (North East 2,584 kWh, Wales 2,700 kWh, Yorkshire 2,721 kWh) have small effects against the majority of other regions except each other, apart from Yorkshire and North East, which have a small effect. These three regions form the low-consumption group.

Regional variation is most pronounced for gas consumption metrics (gas EUI and average gas per meter), where London consistently ranks highest, and the South West ranks lowest, with medium-to-small effect sizes. Since gas consumption is temperature-normalised by region, these differences are unlikely to reflect climatic heating demand. The effect sizes indicate that the high-consumption groups (London and Yorkshire for gas EUI, London alone for Avg. gas) are significantly separated from the lowest-consumption groups (medium effect sizes against Wales and the South West for gas EUI, and South West for Avg Gas) but differ only slightly (small effect or no effect sizes) from the medium-consumption groups.

Regional variation in electricity highlights the South East and London as ranking highly for electricity EUI and average electricity, while Wales and the North East are consistently low. For electricity EUI, the regions are less strongly separated, with small effects only distinguishing the low consumption groups (Wales and the North East). When looking at average electricity per meter the distinction is stronger, with the North East more significantly underconsuming (medium effect sizes) against the top consuming group (South East, London and East of England).

The difference between the EUI metrics and the average per meter metrics reflects that buildings can have more than one meter, and in London, the average meters per building is higher than the national average (mean of 4.6 gas meters per building vs. the national mean of 2). This increased number of gas meters per building reflects larger buildings but lower building count in London, whilst the number of meters per gross internal area for London is on par with the national mean (London mean 1.3e-2 vs. national mean of 1.3e-2 gas meters per m^2^)

After regional investigations, we conclude that London does show some small differences in behaviour to other regions but it is not a significant outlier. London is a consistent top consumer across all energy metrics but the differences, while statistically significant, are generally small in effect sizes, apart from with respect to the South West and Wales for gas metrics and the North East and Wales for electricity. The consistency in the energy performance of London when filtering on the confidence metric ensures that any bias in the area derivation that leads to higher Morris parameters in London does not impact the validity of the energy results and the extreme results for London Morris results are a feature of thresholds used in the derivation.

### Limitations

Several key limitations of this dataset, although mentioned within this paper, are worth highlighting explicitly. Firstly, the geographic coverage is not complete. Within England and Wales postcodes are excluded that are not wholly domestic based on typology of the buildings within. This excludes mixed-use postcodes, which tends to bias to towards exclusions in city centres, as seen in Fig. 3(b) with notable higher exclusions in central London. Postcodes with no gas connections are also excluded, with postcode exclusions occurring when postcodes do not have gas and electricity data. This causes exclusions of some rural communities where no gas connections exist, and can be noted in parts of Wales in Fig. 3(b).

Secondly, the energy data used, as published in the Subnational Consumption statistics, is not the same as smart meter data despite being called ‘real’ energy data. There is still an element of modelling involved, as noted in Calderon *et al*.^31^, the method for calculating energy consumption data includes processing ‘Annual Quantity’, in which two meter readings taken 6-18 months apart are used, and used to calculate the amount used by that meter in one year (DESNZ^32^). The authors argue that this data should be considered as not purely empirical but as a mix between empirical and modelled. The electricity consumption consists of approximately 80% actual readings and 20% estimated readings.

## Data Availability

The dataset is available at APOLLO, University of Cambridge Repository https://doi.org/10.17863/CAM.113850.2. Two datasets (Dataset 1 and Dataset 2) as defined in the Data Records section are given, stored in two comma-separated value (CSV) files, indexed by postcode. The file NEBULA_englandwales_domestic_filtered.csv contains Dataset 1, the processed NEBULA data, including the filters and validations given in the above sections, to offer a clean sample for energy modelling. The citation for this dataset is^33^. Dataset 2, the full set of domestic data without filters is given in NEBULA_englandwales_domestic_unfiltered.csv. The citation for this dataset is^34^. The full index of column names and data types is provided in a separate metadata table in the APOLLO repository. The data is provided in CSV form for ease of use.

## Code availability

The code for generating the dataset is available at https://github.com/sdgresearch/NebulaStockModel. Repo uses the Python programming language and contains details on the appropriate environment. A Read Me offers information on running the code (locally or on a high-performance computing (HPC) machine) and the repository structure.
